# The safety and efficacy of human umbilical cord mesenchymal stem cell for acute respiratory distress syndrome: an open-label and multicenter phase 1 clinical trial

**DOI:** 10.3389/fimmu.2026.1848989

**Published:** 2026-06-10

**Authors:** Qinggang Ge, Libo Zheng, Man Zhao, Chao Li, Zhiling Zhao, Zongyu Wang, Qiang Zhang, Mai Shi, Yuxuan Li, Jianan Zhang, Yixian Qiao, Senhao Wei, Ning Shen, Haomiao Long, Yongjun Liu, Jie Qiao

**Affiliations:** 1 Department of Intensive Care Medicine, Peking University Third Hospital, Beijing, China; 2 Stem Cell Biology and Regenerative Medicine Institution, Yi-Chuang Institute of Bio-Industry, Beijing, China; 3 Department of Pulmonary and Critical Care Medicine, Peking University Third Hospital, Beijing, China; 4 State Key Laboratory of Female Fertility Promotion, Center for Reproductive Medicine, Department of Obstetrics and Gynecology, Peking University Third Hospital, Beijing, China

**Keywords:** acute respiratory distress syndrome - ARDS, anti-inflammation, clinical trial, dose escalation, human umbilical cord mesenchymal stem cells, immunoregulation, multicenter, phase 1

## Abstract

**Trial design:**

Acute Respiratory Distress Syndrome (ARDS) remains a life-threatening critical illness with high mortality and limited specific therapies. This open-label, multicenter Phase I clinical trial aimed to evaluate the safety, tolerability, and preliminary efficacy of allogeneic human umbilical cord mesenchymal stem cells (hUC-MSCs, BC-U001) in patients with mild-to-moderate ARDS. A total of 12 eligible patients were enrolled into three dose groups following a “3 + 3” dose-escalation design from 2019 to 2024.

**Methods:**

All patients received standard ARDS care plus a single intravenous infusion of BC-U001, with 28-day follow-up to assess safety and efficacy as well as exploratory immunological indicators (immunoglobulins, inflammatory cytokines, lymphocyte subsets).

**Results:**

Baseline characteristics were balanced across groups except for more severe baseline lung injury in high dose patients (*P = 0.027*). No dose-limiting toxicity, treatment-related severe adverse events (SAE), or Suspected Unexpected Serious Adverse Reactions (SUSAR) were observed across all dose groups. The overall 28-day all-cause mortality rate was 8.3% (1/12), with 0% mortality among 5 COVID-19-related ARDS patients. Efficacy analysis showed significant improvements in the middle dose group, including a marked increase in PaO_2_/FiO_2_ (+100.07 mmHg, *P < 0.001*) and PaO_2_ (+21.88 mmHg, *P = 0.002*), as well as significant reductions in Lung Injury Score (LIS), Sequential Organ Failure Assessment (SOFA), and Acute Physiology and Chronic Health Evaluation (APACHE) II scores, without dose-dependent effects observed. Exploratory analyses preliminarily revealed that hUC-MSCs modulated the inflammatory response and restored immune balance.

**Conclusions:**

These findings demonstrate that hUC-MSCs are safe and well-tolerated in mild-to-moderate ARDS patients, with the middle dose showing promising therapeutic effects. This trial provides critical data to support the design of future large-scale, randomized controlled trials to confirm the efficacy of hUC-MSCs for ARDS.

## Introduction

ARDS is one of the most challenging critical conditions in intensive care, triggered by multiple factors. Primary causes include pneumonia, extrapulmonary sepsis, trauma, chemical toxicity, and fat embolism. According to the Berlin definition, ARDS is classified by oxygenation index into mild (200 ~ 300 mmHg), moderate (100 ~ 200 mmHg), and severe (< 100 mmHg), with mortality rates increasing accordingly (1, 2). The epidemiological characteristics illustrated that its incidence rate among intensive care unit (ICU) patients was approximately 10%, accounting for 23% of all mechanically ventilated patients, with the overall mortality rate remaining at around 40% (3, 4). Despite the widespread use of strategies such as low tidal volume ventilation, prone position ventilation, and extracorporeal membrane oxygenation (ECMO), there is currently a lack of specific therapeutic approaches due to the pathological complexity and heterogeneity of the disease (5). Additionally, the treatment costs for ARDS are prohibitively high, imposing a significant economic burden on individual families and public health systems. In the United States, ARDS patients typically undergo prolonged hospital stays and consume substantial medical resources. Many patients require significant follow-up care after discharge, further escalating healthcare expenditures (6). Both the incidence of ARDS and the demand for related medical resources increased significantly during the COVID-19 pandemic, further straining public health systems (5). Confronted with unmet medical needs, it is imperative to develop novel therapeutic option that target cytokine storms and immune dysregulation-mediated excessive inflammatory responses, accelerate the recovery of lung tissue function, and reduce patient mortality.

The potential mechanisms and current clinical applications of mesenchymal stem cells (MSCs) in the treatment of ARDS have emerged as a hotspot in recent years, owing to their multifaceted anti-inflammatory, immunomodulatory, and tissue repair capabilities (7–9). The meta-analysis demonstrated that MSC treatment significantly reduces mortality rates in ARDS patients and improves oxygenation indices (10, 11). Moreover, the application of MSCs in COVID-19-related ARDS has demonstrated positive effects, including attenuation of the inflammatory response and modification of pulmonary function (12, 13). Notably, the source and preparation method of MSCs may influence their therapeutic efficacy. Studies comparing the effects of MSCs from different sources in ARDS treatment have found that hUC-MSCs demonstrate superior performance in improving animal survival rates and reducing pulmonary inflammation (14). They are extracted from discarded umbilical cords and rapidly expanded to clinical quantities, with lower Class I/II human leukocyte antigen, which could help reduce immunogenicity (15). We are scaling up production of hUC-MSCs compliant with the European Union’s Good Manufacturing Practice (GMP) standards to support upcoming multicenter clinical trials, which will require substantial cell doses.

However, several large-scale clinical trials of mesenchymal stem cell (MSC) therapy for ARDS, including the Mesoblast trial (NCT04371393) (16) and the STAT study(NCT03818854) (17), reported neutral or negative results. In December 2020, Mesoblast voluntarily terminated its Phase 2/3 trial for COVID-19-associated ARDS ahead of schedule, following the recommendation of the Data Safety Monitoring Board (DSMB), as the trial was unlikely to meet its primary endpoint (a 43% reduction in 30-day mortality) by the time the planned 300 patients had been enrolled. Although the trial was terminated early, Mesoblast completed follow-up of enrolled patients as recommended by the DSMB and observed the following results: a 14% reduction in 60-day mortality in the overall population, though this was not statistically significant; Patients < 65 years of age: a 46% reduction in 60-day mortality (26% vs. 42%, HR 0.54, P = 0.0485) and a 48% reduction in 90-day mortality (HR 0.52, P = 0.038); In patients < 65 years of age receiving dexamethasone, remestemcel-L reduced 60-day mortality by 75% (P = 0.0037). The STAT study ultimately enrolled 101 patients with COVID-19-associated ARDS and 19 patients with non-COVID classic ARDS; moreover, there was a significant imbalance in the randomization of these 19 patients (only 3 in the MSC group and 16 in the placebo group). Possible reasons for the failure include: significant differences in baseline plasma levels of markers such as Ang-2, IL-6, and IL-8 in COVID-19 patients compared to those with classic ARDS, which may have weakened the reparative potential of MSCs; following the RECOVERY trial, approximately 80% of COVID-19 patients received dexamethasone, and its anti-inflammatory effects may have masked or offset the effects of MSCs; *Post-hoc* analyses suggest that COVID-19 subtypes defined by baseline plasma biomarkers (Class 1 vs. Class 2) exhibit markedly different responses to MSCs — 60-day mortality actually increased in Class 2 patients following MSC administration, whereas it decreased in Class 1 patients (17), indicating significant treatment heterogeneity. Therefore, these do not mean that MSCs have no potential in the treatment of ARDS.

It should be emphasized that these studies used cryopreserved MSC products, which have inherent limitations: the cell viability after thawing, residual cryoprotectant dosage, and batch-to-batch consistency are difficult to strictly control, which may compromise therapeutic efficacy and safety. In contrast, BC-U001 used in this study is a fresh preparation (valid for 24 hours after preparation), produced in a GMP workshop compliant with European Union standards, and has obtained the Stem Cell Preparations Quality Management Qualification Certificate issued by the Chinese Society for Biomedical Engineering. The entire production, quality inspection and release process follows strict standard operating procedures (SOPs), ensuring stable and controllable cell quality, potency and consistency. We have established a national cold-chain logistics system that guarantees delivery to any clinical center within 24 hours, ensuring that patients receive fresh and active cell products within the valid period. These critical differences in product form, production process and quality control may explain the distinct efficacy outcomes between our study and previous large-scale negative trials.

Preclinical studies of BC-U001 demonstrated robust anti-inflammatory, immunomodulatory, and lung-protective effects in ARDS rats, with no obvious toxicity or adverse reactions observed at the tested doses, supporting its clinical development for ARDS (18).The objective of this study was to establish safety and explore efficacy of allogenic hUC-MSCs infusions in hospitalized patients with ARDS. Here we report the results at 1 month of follow-up of a multicenter phase 1 clinical trial testing this cell-based therapy approach.

## Results

### Patients screened

**Figure 1:**
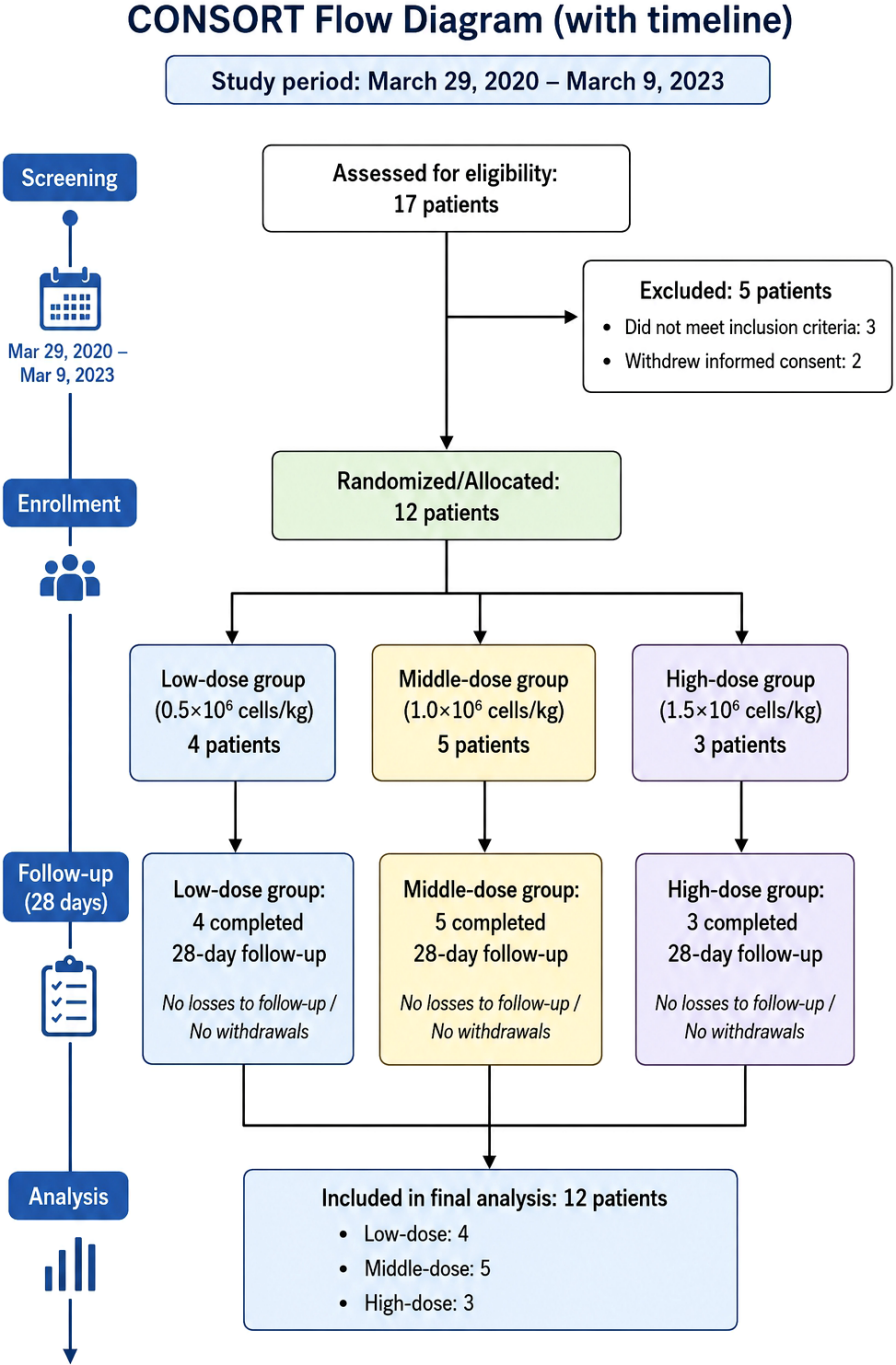
CONSORT flow diagram. The trial enrolled patients between March 29, 2020 and March 9, 2023. A total of 17 patients were screened, 5 were excluded, and 12 were allocated to three dose groups. All 12 patients completed the 28-day follow-up and were included in the final analysis.

Between March 29, 2020 and March 9, 2023, a total of 17 patients were screened for eligibility. Five patients were excluded: 3 did not meet the inclusion criteria and 2 withdrew informed consent. The remaining 12 patients were enrolled and allocated to three dose groups: 4 in the low-dose group, 5 in the middle-dose group, and 3 in the high-dose group. All 12 patients completed the 28-day follow-up without loss to follow-up or dropout, and all were included in the final analysis (**Figure 1**).

### Baseline data

All three patient groups showed no statistically significant differences in age, gender distribution, or BMI (*P > 0.05*), indicating balanced baseline demographic characteristics (**Table 1**). Similarly, no differences were observed in the distribution of oxygenation index (PaO_2_/FiO_2_) and ARDS severity (mild/moderate) among the three groups at baseline (*P = 0.760, P = 0.902*) (**Table 1**), indicating comparable baseline pulmonary function impairment across all three groups. It also excluded the interference of comorbidities on subsequent efficacy evaluation (*P > 0.05*) (**Table 1**). Notably, all patients (100%) in the low-dose and medium-dose groups had LIS scores ranging from 0.25 to 2.5, whereas only 33.3% (1/3) of patients in the high-dose group fell within this range (**Table 1**). A significant 66.7% (2/3) of patients in the high-dose group had LIS scores exceeding 2.5 (*P = 0.027*) (**Table 1**). The potential impact of this baseline disparity must be considered in subsequent efficacy analyses. Intergroup comparisons should utilize “change from baseline” measures to mitigate the confounding effect arising from this baseline imbalance in lung injury severity.

**Table 1:** Baseline characteristics by dose group.

Characteristics	Total (n = 12) n (%)	Low dose (n = 4) n (%)	Middle dose (n = 5) n (%)	High dose (n = 3) n (%)	*P*
Sex, n (%)					0.350
Male	8 (66.7)	2 (50.0)	3 (60.0)	3 (100.0)	
Female	4 (33.3)	2 (50.0)	2 (40.0)	0 (0.0)	
Age, mean ± SD, years	55.25 ± 17.72	57.25 ± 21.00	49.00 ± 20.37	63.00 ± 6.24	0.226
Race, n (%)					>0.05
Han	12 (100.0)	4 (100.0)	5 (100.0)	3 (100.0)	
Other	0 (0.0)	0 (0.0)	0 (0.0)	0 (0.0)	
PaO_2_/FiO_2_ ratio at enrollment, median (IQR)	192.6 (161.9-262.9)	234.0 (178.6-280.1)	194.2 (168.7-212.8)	189.5 (159.9-243.4)	0.760
ARDS severity stratification, n (%)					0.902
Mild	5 (41.7)	2 (50.0)	2 (40.0)	1 (33.3)	
Moderate	7 (58.3)	2 (50.0)	3 (60.0)	2 (66.7)	
BMI, mean ± SD, kg/m^2^	24.84 ± 4.40	23.92 ± 3.48	25.08 ± 5.80	25.66 ± 4.25	0.550
Lung injury score, n (%)					0.027
0.25-2.5	10 (83.3)	4 (100.0)	5 (100.0)	1 (33.3)	
>2.5	2 (16.7)	0 (0.0)	0 (0.0)	2 (66.7)	
Smoker (former), n (%)	4 (33.3)	1 (25.0)	3 (60.0)	0 (0.0)	0.199
Comorbidities, n (%)					
Diabetes	4 (33.3)	2 (50.0)	1 (20.0)	1 (33.3)	0.638
Hypertension	7 (58.3)	2 (50.0)	3 (60.0)	2 (66.7)	0.902
Obesity (BMI > 30)	1 (8.3)	0 (0.0)	1 (20.0)	0 (0.0)	
Heart disease	5 (41.7)	2 (50.0)	1 (20.0)	2 (66.7)	0.396

### Overall outcome and assessment

The LUNG SAFE study reported a 28-day all-cause mortality rate of 34.9% among ARDS patients, with approximately 29% for mild cases and approximately 35% for moderate cases (19). In this trial, all subjects survived except for one case who died after being discharged against medical advice when family members declined further treatment despite improved oxygenation index. The observed 28-day all-cause mortality rate was 8.3% (**Table 2**). Among the 5 patients with COVID-19-related ARDS, all survived, with an observed 28-day all-cause mortality rate of 0% (**Table 2**).

**Table 2:** Summary of all adverse events for enrolled subjects.

Number	Etiology	Dose	Death (yes = 1/No = 0)
01001	COVID-19	Low	0
01002	COVID-19	Middle	0
D001	Severe pneumonia	Low	0
D002	Severe pancreatitis	Low	0
D003	Multiple fracture	Low	0
Z001	Severe pneumonia	Middle	1
Z002	Severe pancreatitis	Middle	0
Z003	A/H1N1 influenza	Middle	0
Z004	Pulmonary contusion	Middle	0
G001	COVID-19	High	0
G002	COVID-19	High	0
G003	COVID-19	High	0

### Adverse events

Three patients in the low dose group experienced 31 AE (patient incidence rate: 75.0%), including 14 mild events (45.2%), 14 moderate events (45.2%), and 3 severe events (9.7%) (**Table 3**). One patient experienced 3 SAE (patient incidence rate 25.0%), all occurring at the final follow-up: multiple fractures, paroxysmal ventricular tachycardia, and seizure (**Table 3**). All were deemed unrelated to treatment. Five patients in the middle dose group experienced 36 AE (patient incidence rate 100.0%), including 14 mild events (38.9%), 21 moderate events (58.3%), and 1 severe event (2.8%) (**Table 3**). One patient experienced one SAE (patient incidence rate 20.0%), occurring 14 days post-treatment (**Table 3**), resulted in death due to severe pneumonia. The high dose group reported 36 adverse events (AE) in 3 patients (incidence rate 100.0%), including 20 mild events (55.6%) and 16 moderate events (44.4%) (**Table 3**). No treatment-related AE/SAE and SUSAR were observed in any group, consistent with the known safety profile of hUC-MSCs in the treatment of respiratory diseases, suggesting the risks are manageable.

**Table 3:** Summary of all AE for enrolled subjects.

Topics	Low dose (n = 4), n (%)	Middle dose (n = 5), n (%)	High dose (n = 3), n (%)
Number of AE reported	31	36	36
Number of subjects with AEs	3	5	3
Number of SAE reported	3	1	0
Number of subjects with SAE	1	1	0
Number of AE by severity			
Mild	14 (45.2)	14 (38.9)	20 (55.6)
Moderate	14 (45.2)	21 (58.3)	16 (44.4)
Severe	3 (9.7)	1 (2.8)	0 (0.0)
Subjects with AE by severity			
Mild	3 (75.0)	4 (80.0)	3 (100.0)
Moderate	2 (50.0)	3 (60.0)	3 (100.0)
Severe	1 (25.0)	1 (20.0)	0 (0.0)
Number of AE related to treatment			
Definite	0 (0.0)	0 (0.0)	0 (0.0)
Probable	0 (0.0)	0 (0.0)	0 (0.0)
Possible	2 (6.5)	3 (8.3)	2 (5.6)
Unlikely	29 (93.5)	32 (88.9)	34 (94.4)
Unrelated	0 (0.0)	1 (2.8)	0 (0.0)
Subjects with AE related to treatment			
Definite	0 (0.0)	0 (0.0)	0 (0.0)
Probable	0 (0.0)	0 (0.0)	0 (0.0)
Possible	2 (50.0)	3 (60.0)	2 (66.7)
Unlikely	3 (60.0)	4 (80.0)	3 (100.0)
Unrelated	0 (0.0)	1 (20.0)	0 (0.0)

### The survival rate without SAE

**Figure 2:**
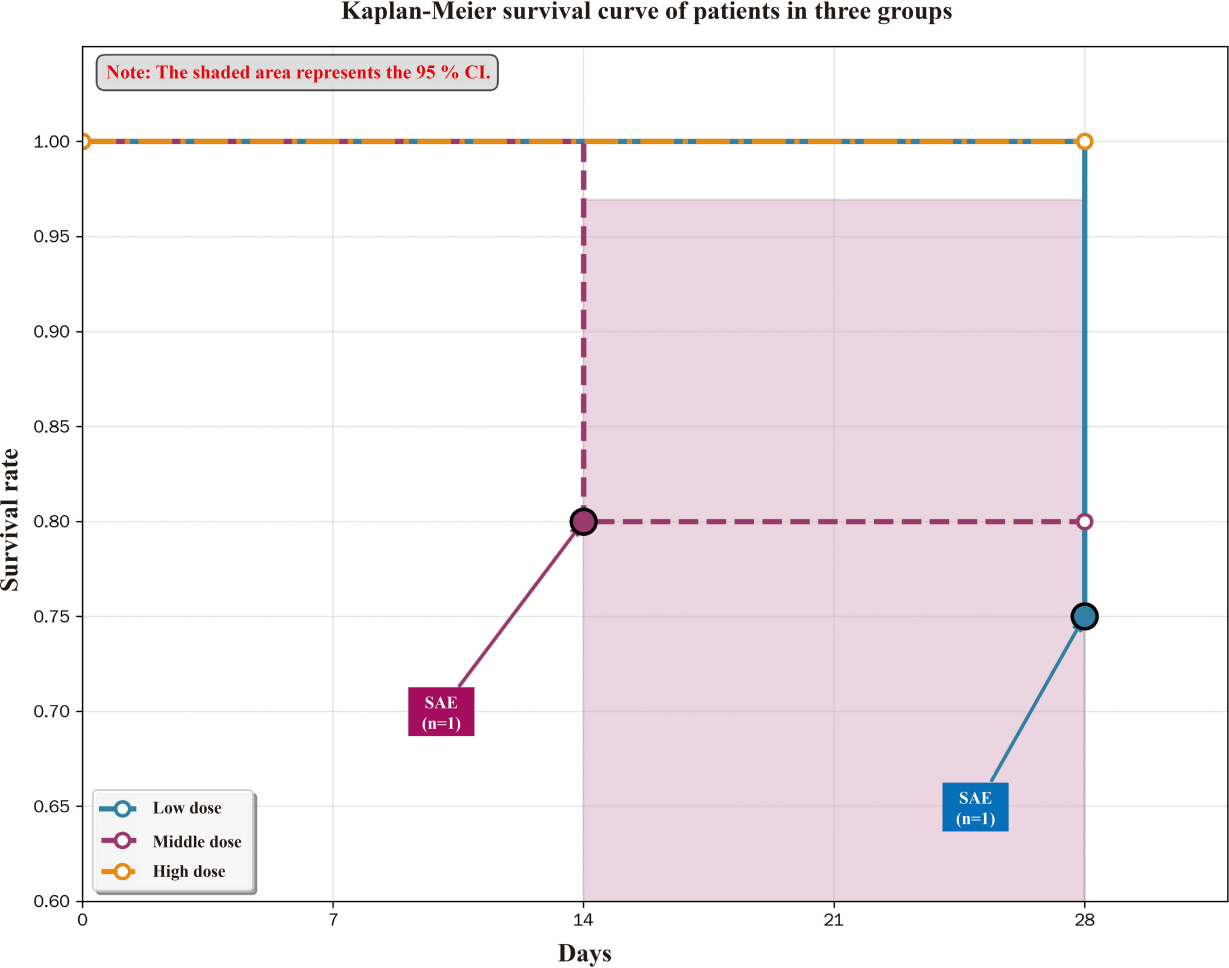
The KM-curve of SAE-free survival rate in three groups. The survival curve for the high-dose group remained consistently at 1.0 (100% survival rate) without decline. The medium-dose group maintained an 80% survival rate after experiencing one SAE at day 14. The low-dose group maintained a 75% survival rate after experiencing one SAE at day 28.

One subject in the low dose group experienced an SAE at the final follow-up visit, with an SAE-free survival rate of 75% (**Figure 2**). A single SAE was recorded in the middle dose group at Visit 9, with an SAE-free survival rate of 80% (**Figure 2**). The survival curve for the high-dose group was flat and linear, with a 100% survival rate for patients without SAE (**Figure 2**). The differences among the three groups were not statistically significant because of the limited sample size (**Supplementary Table 1**). From an overall survival perspective, it is recommended to enhance close monitoring (e.g., vital signs, organ function) within the first two weeks following treatment.

### Efficacy evaluation

**Figure 3:**
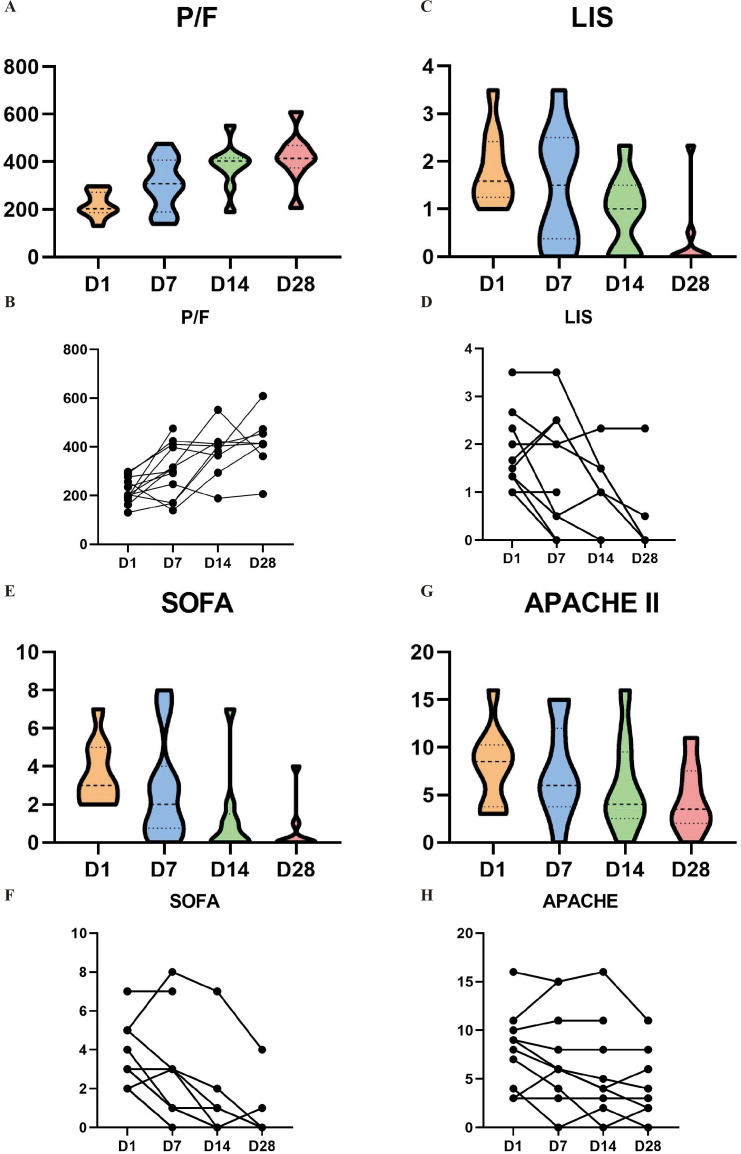
The hUC-MSCs alleviated lung injury and improved pulmonary function in patients with mild-to-moderate ARDS. **(A)** The violin plot depicting the distribution of oxygenation indices at each visit point for all subjects except for dropouts. **(B)** The trends in oxygenation index for all subjects except for dropouts on Day 1, Day 14 and Day 28. **(C)** The violin plot depicting the distribution of LIS at each visit point for all subjects except for dropouts. **(D)** The trends in LIS for all subjects except for dropouts on Day 1, Day 14 and Day 28. **(E)** The violin plot depicting the distribution of SOFA score at each visit point for all subjects except for dropouts. **(F)** The trends in SOFA score for all subjects except for dropouts on Day 1, Day 14 and Day 28. **(G)** The violin plot depicting the distribution of APACHE II at each visit point for all subjects except for dropouts. **(H)** The trends in APACHE II for all subjects except for dropouts on Day 1, Day 14 and Day 28.

Overall, oxygenation indices increased in all three groups of subjects following treatment (**Figure 3A**), with the majority of significant improvements observed by Day 14 (**Figure 3B**). The opposite trends were observed in scores related to lung tissue injury and function, including LIS (**Figures 3C, D**), SOFA (**Figures 3E, F**), and APACHE II (**Figures 3G, H**). The middle-dose group showed statistically significant improvements in PaO_2_/FiO_2_ and PaO_2_ (**Supplementary Table 2–3**). However, no significant dose-dependent changes in oxygenation index, arterial oxygen partial pressure, and lactate levels were observed across the three groups before and after treatment (**Supplementary Table 2–4**). Similarly, statistically significant differences were found in reductions of LIS, SOFA, and APACHE II scores in the middle-dose group (**Supplementary Table 5–7**). These exploratory results suggest that intravenous infusion of 1.0×10 (6) cells/kg may yield promising preliminary therapeutic signals on pulmonary function and tissue injury in patients with mild to moderate ARDS, supporting its selection as the target dose for subsequent confirmatory clinical trials.

### The anti-inflammatory and anti-infective effects of hUC-MSC

**Figure 4:**
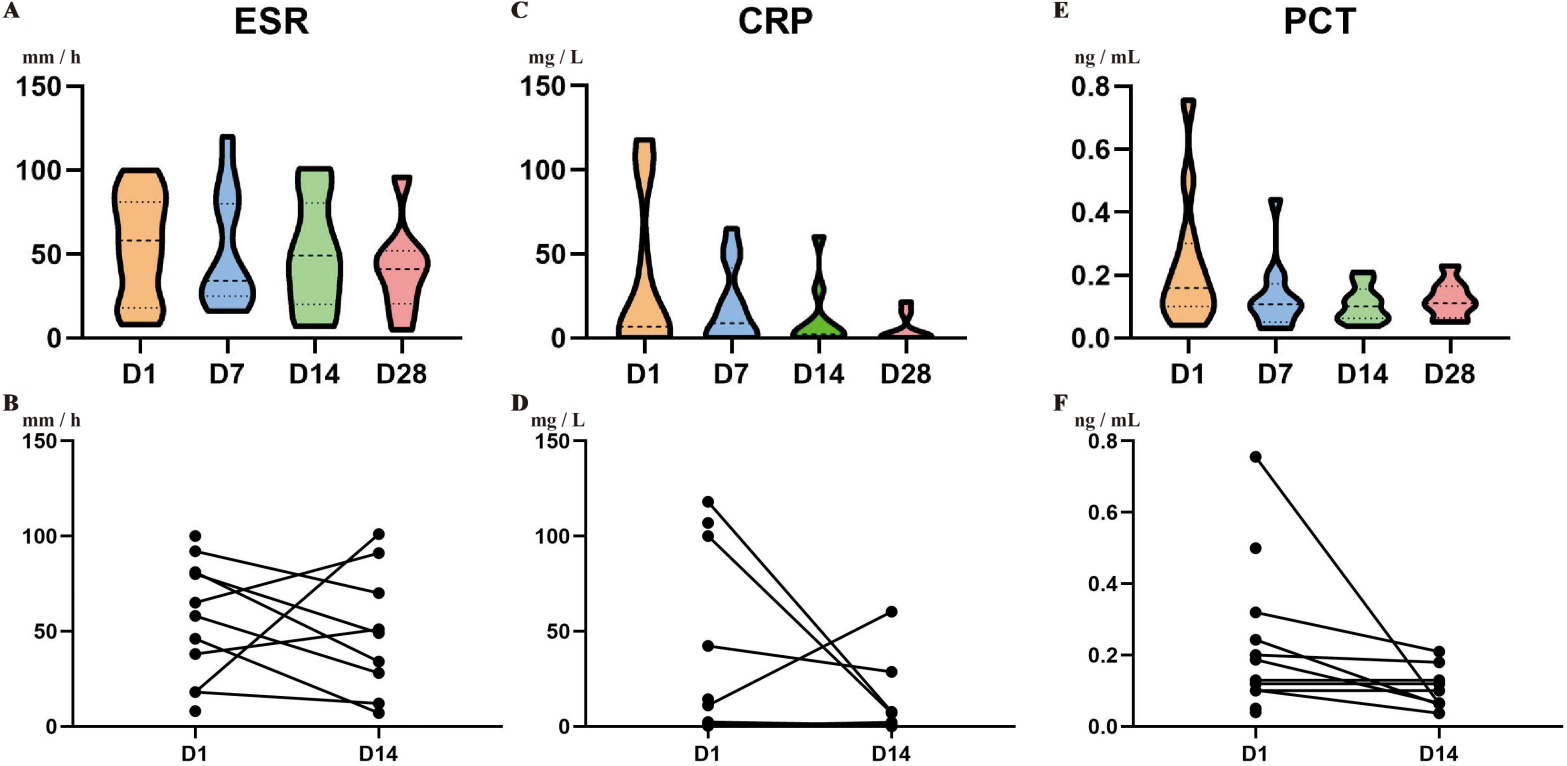
Modulation of inflammatory response and infection by hUC-MSCs in ARDS. **(A)** The violin plot depicting the distribution of ESR level at each visit point for all subjects except for dropouts. **(B)** The trends in ESR level for all subjects except for dropouts on Day 1 and Day 14. **(C)** The violin plot depicting the distribution of CRP level at each visit point for all subjects except for dropouts. **(D)** The trends in CRP level for all subjects except for dropouts on Day 1 and Day 14. **(E)** The violin plot depicting the distribution of PCT at each visit point for all subjects except for dropouts. **(F)** The trends in PCT level for all subjects except for dropouts on Day 1 and Day 14.

To preliminarily investigate the potential mechanisms of hUC-MSCs in treating ARDS at the clinical stage, we measured ESR, CRP, PCT, and a series of cytokine levels at each follow-up point for patients. All patients showed a downward trend in ESR, CRP and PCT levels following treatment (**Figure 4**), consistent with the anti-inflammatory and potential anti-infective effects of hUC-MSCs. Individual variability was observed, likely reflecting the inherent heterogeneity of ARDS. Additionally, changes in cytokine levels reflect the dynamic alterations of inflammatory state. It was shown that IL-6 and IL-8 levels in subjects exhibited a downward trend within 14 days, even though cytokine levels at each visit point showed no significant differences (**Supplementary Figures 2A–E**). Interestingly, changes of IL-6 and IL-10 in each group showed opposite trends (**Supplementary Figures 2F, G**, **Supplementary Table 8**), with a decrease in their ratio observed (**Supplementary Figure 2H**, **Supplementary Table 8**). These preliminary evidences suggest that hUC-MSCs may exert anti-inflammatory effects in the treatment of ARDS.

Logically, Logically, ARDS patients exhibit significantly elevated CRP levels due to cytokine storms (20). In the COVID-19-ARDS cohort, ICU mortality was significantly higher in the “persistently elevated” CRP group compared to the “decreasing or normal” group during hospitalization (62.7% vs 18.6%, *P < 0.00001*), suggesting that sustained elevation may indicate secondary infection or excessive inflammation (21). Participants in this trial generally exhibited a downward trend in CRP levels (**Figures 4C, D**), without statistically significant differences due to the small sample size. This finding is consistent with current research results, suggesting the anti-infective effects of hUC-MSCs, particularly secondary infections. Current evidence most strongly supports the use of PCT for “differentiating infection” and “assessing the severity of ARDS driven by infection.” Within 72 hours of admission, serum PCT levels in the sepsis-ARDS group were significantly higher than those in the non-infectious-ARDS group, with minimal overlap between the two groups (22). Furthermore, the more severe the bacterial infection (such as septic shock or multiple organ failure), the higher the peak PCT level (22). If treatment is effective, PCT declines much more rapidly than CRP, making it useful for assessing the efficacy of anti-infective therapy and predicting prognosis (22). All patients demonstrated a degree of PCT decline, although individual variations inevitably occurred given the heterogeneity of the disease (**Figures 4E, F**). This further suggests the potential anti-infective effects of the investigational drug in this trial.

### The immunomodulatory effects of hUC-MSC in the treatment of ARDS

Further analysis revealed changes in the patient’s immune system following cell infusion. All immunoglobulins remained within normal clinical ranges, showing neither excessive activation (e.g., elevated IgE) nor suppression (e.g., decreased IgG) (**Supplementary Figures 3A–D**). The complement system exhibited moderate activation (no significant differences in C3 and C4, elevated CH50, **Supplementary Figures 3E–G**). Overall, a balanced state of “stable IgG^+^ reduced IgE^+^ moderately activated complement” was observed, preventing tissue damage caused by excessive immune responses. This aligns with the regulatory characteristics of hUC-MSCs, which “enhance defense without inducing inflammation”.

**Figure 5:**
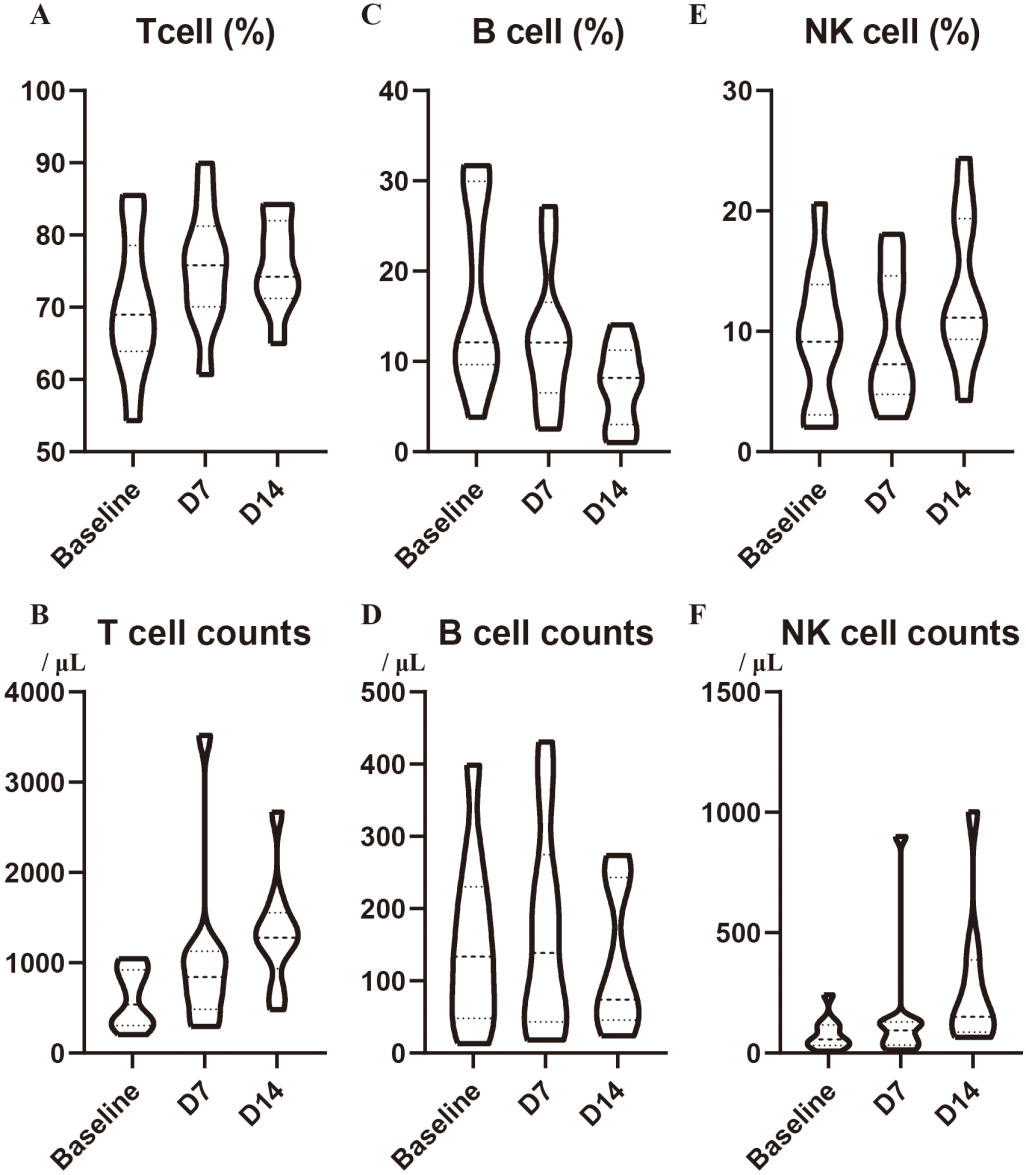
Changes in immune cells. **(A, C, E)** The percentage of T cells, B cells, and NK cells in all subjects except for dropouts from baseline to Day 14, separately. **(B, D, F)** The counts of T cells, B cells, and NK cells in all subjects (units: cells/μL, equivalent to cells/mm3) except for dropouts from baseline to Day 14, separately.

Notably, a proportion of patients exhibited lymphopenia at baseline, which is a well-recognized common complication and poor prognostic factor in ARDS, particularly in COVID-19-related ARDS (23). Following hUC-MSC infusion, T cells and NK cells showed a trend of increase at Day 7 and Day 14, while B cell counts remained relatively stable (**Figure 5**). No significant decrease in any lymphocyte subset was observed at any time point after treatment, indicating that hUC-MSC therapy did not induce systemic immunosuppression. This finding is consistent with a recent study reporting that MSCs can promote the resolution of lymphopenia in COVID-19 ARDS patients by modulating immune cell homeostasis (23). Collectively, these results demonstrate that hUC-MSCs exert beneficial immunomodulatory effects without causing immune suppression, further supporting their favorable safety profile in ARDS treatment.

## Discussion

This multicenter, open-label, dose-escalation phase I trial evaluated the safety and preliminary efficacy of fresh allogeneic hUC-MSCs (BC-U001) in patients with mild-to-moderate ARDS. The primary objective of establishing a safe dose range (0.5 × 10^6^ to 1.5 × 10^6^ cells/kg) was successfully achieved, with no treatment-related serious adverse events observed. The observed 28-day all-cause mortality rate in this trial was 8.3%. Furthermore, the investigational drug showed promising preliminary signals of improvement in lung tissue injury and functional scores in exploratory analyses. Mechanistic findings also demonstrated the anti-inflammatory and immunomodulatory potential of hUC-MSCs in ARDS.

The safety and efficacy of hUC-MSCs in the treatment of ARDS have become a hot topic of research in recent years. Our study confirmed the favorable safety profile of hUC-MSCs, with no treatment-related SAEs observed, which aligns with previous systematic reviews demonstrating the safety of MSC therapy in ARDS (10). In patients with COVID-19-induced ARDS, the application has also demonstrated favorable safety and tolerability, with no serious adverse events observed (24, 25). In this study, only a few mild/moderate AE “possibly related” to treatment were observed in the 12 patients (6.5% in the low dose group, 8.3% in the middle dose group, and 5.6% in the high-dose group), consistent with existing clinical evidence on MSCs therapy for ARDS. Our research further supplement safety data across the dose range: within the 0.5×10^6^ to 1.5×10^6^ cells/kg dose interval, the high-dose group (1.5×10^6^ cells/kg) exhibited a 0% SAE incidence rate, and the severity of adverse events did not increase with higher doses. This provides direct evidence for subsequent clinical dose selection.

The administration of hUC-MSCs has also demonstrated positive effects in improving lung function and overall prognosis in ARDS patients. In COVID-19-related ARDS patients, infusion of hUC-MSCs significantly improved patients’ oxygenation indices and pulmonary inflammation (26). We were encouraged to find that the oxygenation index in the middle-dose group patients significantly improved from baseline, while LIS, SOFA, and APACHE II scores all decreased significantly. Similar to Monsel et al. (27), we noted improvements in oxygenation and reduced injury scores in the middle-dose group, supporting a potential therapeutic signal. In contrast, large randomized trials using cryopreserved MSCs, such as the STAT study (17), reported neutral efficacy outcomes, which may be related to differences in cell product quality and preparation methods. Notably, 66.7% of patients in the high-dose group had LIS scores > 2.5 (significantly higher than other groups), indicating more severe lung injury that may have partially offset therapeutic effects. Future trials should employ stratified randomization to reduce baseline confounding. It has also been reported that hUC-MSCs can alleviate ARDS symptoms by regulating immune cells and suppressing the release of inflammatory cytokines (28). We also observed downregulation of proinflammatory cytokines such as IL-6 and IL-8, and upregulation of anti-inflammatory cytokines such as IL-10 following cell infusion. T cell and NK cell counts showed a slight increase 7 to 14 days after treatment, accompanied by decreased IgE levels and moderate complement system activation. These exploratory findings generate a hypothesis that hUC-MSCs may exert immunomodulatory effects in ARDS, which requires confirmation in larger controlled trials.

All 5 patients with COVID-19-related ARDS in this study survived (28-day mortality rate 0%), significantly lower than the 35% mortality rate reported for moderate ARDS in the LUNG SAFE study (19). The 0% mortality rate observed in our small cohort of COVID-19-related ARDS patients warrants further investigation in a larger, controlled population. Cytokine storms and immune dysregulation are hallmark features of this subtype. Recent report has confirmed that activated caspase-1, IL-1β, and IL-6 are significantly elevated in the lungs of COVID-19 patients (29). Additional research indicated that the SARS-CoV-2 virus exacerbates cytokine storms by downregulating the ACE-2 signaling pathway, promoting inflammatory responses, and causing the immune system to fail in transitioning from innate to adaptive immunity (30). These findings corroborate the observed decrease in inflammatory markers and increase in immune cell counts among COVID-19 patients in this study. However, we observed a certain degree of elevation in TNF-α following treatment. A clinical study indicates that hUC-MSCs exhibit potent anti-inflammatory effects through the expression and release of soluble TNF receptor 2 (sTNFR2), which correlates with significant clinical improvement (31). Therefore, the potential of TNF-α as a therapeutic target for ARDS warrants further investigation. Nevertheless, these findings collectively reinforce the close association between hUC-MSCs and reduced levels of proinflammatory mediators alongside clinical symptom improvement. Following the COVID-19 pandemic, the need for treating virus-associated ARDS persists. These findings also offer new therapeutic insights for this subgroup, paving the way for subsequent targeted studies with expanded sample sizes.

Most importantly, as a small, open-label phase I trial without a placebo control, all efficacy analyses presented are strictly exploratory and hypothesis-generating in nature, intended to identify potential therapeutic signals and inform future large-scale randomized controlled trials (RCTs). The primary value of this study lies in establishing safety and identifying preliminary signals to guide future trial design. Additionally, this study only followed patients for 28 days and did not evaluate lung function recovery, quality of life, or long-term complications 6 months to 1 year after discharge. Consequently, it is impossible to determine the long-term efficacy and safety of hUC-MSCs. Although trends in inflammatory mediators and immune cells were identified, it remains unclear which indicators could serve as “therapeutic predictive biomarkers” (e.g., whether baseline IL-6 levels influence treatment response), limiting the development of personalized treatment plans. We have obtained CDE approval to conduct a randomized, double-blind, placebo-controlled phase II trial using the middle dose (1.0×10^6^ cells/kg), which will enroll a sufficient number of patients to definitively evaluate efficacy.

## Materials and methods

### Study design

An open-label, “3 + 3” dose-escalation and multicenter Phase I clinical trial has been registered on Clinical Trial Registration and Information Disclosure Platform (https://www.chinadrugtrials.org.cn/index.html) (30/03/2020, CTR20200525) and approved by the Center for Drug Evaluation (CDE) of the National Medical Products Administration (NMPA) (Clinical Trial Drug Approval Number: 2020L00008). The purpose of investigation is to determine the safety of allogeneic hUC-MSCs (BC-U001) and conduct a preliminary assessment potential efficacy in patients with mild-to-moderate ARDS. The protocol was approved by the institutional review board (IRB) of each participating center (Research Ethics Committee of the Academy of Medical Sciences at Peking University Third Hospital; Ethics Committee of Beijing Chaoyang Hospital Affiliated to Capital Medical University; Ethics Committee for Clinical Trials of Drugs and Medical Devices at China-Japan Friendship Hospital; Ethics Committee for Clinical Trials at Quzhou People’s Hospital; Ethics Committee for Clinical Trials at Hebei CNPC Central Hospital). All procedures were performed in accordance with the Declaration of Helsinki and Good Clinical Practice (GCP) guidelines.

### Participants

Eligible participants were male or female patients aged 18 ~ 80 years who met the Berlin definition of mild-to-moderate ARDS. The diagnostic criteria for ARDS (Berlin definition) included: (1) Onset of respiratory symptoms within 1 week of known clinical insult or new/worsening respiratory symptoms; (2) Bilateral opacities on chest imaging that cannot be fully explained by pleural effusion, atelectasis, or lung nodules; (3) Respiratory failure that cannot be fully explained by heart failure or fluid overload, with objective assessment (e.g., echocardiography) required to exclude cardiogenic edema in the absence of risk factors; (4) Oxygenation index (PaO_2_/FiO_2_) of 200 mmHg < PaO_2_/FiO_2_ ≤ 300 mmHg (mild) or 100 mmHg < PaO_2_/FiO_2_ ≤ 200 mmHg (moderate) with positive end-expiratory pressure (PEEP) or continuous positive airway pressure (CPAP) ≥ 5 cmH_2_O.

Participants were included if they met all the above diagnostic criteria, had good compliance to follow the study procedures, and provided written informed consent. Exclusion criteria included: (1) Infections such as hepatitis B, hepatitis C, active or latent tuberculosis, acquired immunodeficiency syndrome (AIDS), syphilis, or other immune system diseases; (2) Severe cardiovascular and cerebrovascular diseases, neuropsychiatric disorders, or hematological abnormalities judged by investigators to increase participant risk; (3) Severe respiratory diseases including severe or very severe chronic obstructive pulmonary disease, severe asthma, pulmonary fibrosis, bronchiectasis, or hemoptysis from other causes; (4) Pulmonary hypertension of WHO functional class III or IV; (5) History of deep vein thrombosis or pulmonary embolism within 6 months before enrollment; (6) Post-lung transplantation status; (7) Severe liver function impairment (aspartate aminotransferase [AST] > 5 times the upper limit of normal); (8) Renal insufficiency (estimated glomerular filtration rate ≤ 50 mL/min/1.73 m^2^) or receipt of continuous renal replacement therapy, hemodialysis, or peritoneal dialysis; (9) Pregnancy, lactation, or positive urine pregnancy test before enrollment, or refusal to use contraception during the study and within 12 months after infusion (except for those who had undergone sterilization or were postmenopausal before screening); (10) History of major surgery or severe trauma within 5 days before enrollment; (11) Use of high-dose corticosteroids (equivalent to methylprednisolone > 240 mg/day) within 3 days before treatment or long-term irregular systemic corticosteroid use for other diseases judged to affect efficacy; (12) Allergy or severe allergic history to low-molecular-weight heparin calcium or human albumin; (13) Participation in other interventional clinical trials or participation in other interventional clinical trials within 3 months before screening; (14) History of malignant tumors; (15) Other factors judged by investigators that may lead to premature termination of the study, such as non-compliance, need for combined treatment for other severe diseases, severe laboratory abnormalities, or family/social factors affecting participant safety or data/sample collection.

### Sample size and dose escalation strategy

A total of 9 ~ 18 participants were planned to be enrolled, divided into three dose groups (low, medium, high), with 3 ~ 6 participants in each group. The dose escalation was conducted following the “3 + 3” rule. The starting dose was set at 0.5×10^6^ cells/kg body weight, the middle dose at 1.0×10^6^ cells/kg body weight, and the maximum escalation dose at 1.5×10^6^ cells/kg body weight, based on previous preclinical and clinical data of hUC-MSCs in immune-related diseases and ARDS.

### Dose escalation strategy

For each dose group, after 3 participants completed 3 days of dose-limiting toxicity (DLT) observation, the next participant in the same group could be enrolled; the next higher dose group could start enrollment only after the previous dose group completed 14 days of DLT observation. If no DLT occurred in 3 participants of a dose group (0/3), the dose could be escalated to the next group. If 1 case of DLT occurred (1/3), 3 additional participants were added to the group; if no DLT occurred in the additional participants (total DLT 1/6), dose escalation was allowed; if ≥ 1 DLT occurred in the additional participants (total DLT ≥ 2/6), dose escalation was terminated, and the previous dose was defined as the maximum tolerated dose (MTD). If no DLT occurred at the maximum dose (1.5×10^6^ cells/kg), this dose was determined as the MTD, and the sponsor and investigators would jointly decide whether to continue dose escalation. DLT was defined as grade ≥ 3 non-hematological toxicity or grade 4 hematological toxicity related to the study drug (according to CTCAE version 5.0).

### Cell isolation

The BC-U001 cells were derived from umbilical cord tissue of healthy donors. Mesenchymal stem cells were obtained using the tissue explant culture method. The detailed procedure is as follows: The umbilical cord was cut into small tissue pieces of approximately 2–3 mm^3^. These tissue pieces were seeded into T75 culture flasks and cultured in DF12 medium (DMEM/F12) supplemented with 10% fetal bovine serum. The cultures were maintained in a humidified incubator at 37°C with 5% CO_2_. The medium was changed every 2–3 days. When cells reached 80 - 90% confluence, they were passaged at a ratio of 1:3. A master cell bank and a working cell bank were established at passage 2 and passage 5, respectively. The BC-U001 cells used for transplantation in this study were all at passage 6 to avoid potential phenotypic or functional changes associated with either too early or too late passaging.

### hUC-MSCs product

The study drug was BC-U001 (hUC-MSCs), a class 1 therapeutic biological product, with the active ingredient being hUC-MSC that met the definition of MSCs by the International Society for Cellular Therapy (ISCT): (1) Adherent growth; (2) Positive expression of CD73, CD90, and CD105 (positive rate ≥ 95%) and negative expression of CD11b, CD19, CD34, CD45, and HLA-DR (positive rate ≤ 2%); (3) Multidirectional differentiation potential into osteoblasts and adipocytes under specific culture conditions. The formulation of BC-U001 was a fresh hUC-MSC suspension with a concentration of 5×10^5^ cells/mL, containing excipients of compound electrolyte injection, 68 IU/mL low-molecular-weight heparin calcium injection, and 1% human albumin, with a specification of 1×10^7^ cells/20 mL (disposable plastic blood bag, 20 mL/bag), stored at 2 ~ 8 °C, and valid for 24 hours after filling.

Quality control methods in addition to phenotypic characterization: In addition to flow cytometry identification of positive (CD73, CD90, CD105) and negative (CD34, CD45, HLA-DR) markers, we performed the following quality control tests: Sterility and endotoxin testing: Bacterial, fungal, and endotoxin levels were tested using pharmacopoeia methods to ensure compliance with transplantation standards. (1) Mycoplasma testing: Culture supernatants were routinely tested using pharmacopoeia methods, and all results were negative. (2) Cell viability: Cell viability was assessed using trypan blue staining before each transplantation, with a required threshold of ≥ 90%. (3) Cell potency assay: Selected batches were tested for their adipogenic, osteogenic, and chondrogenic differentiation capacities, as well as the level of secreted sTNFR1, as biological indicators of potency (32). (4) Karyotype analysis: G-banding karyotype analysis was performed on selected cells at higher passages, and no chromosomal abnormalities were detected.

### Administration

All participants received basic treatment in accordance with the *Guidelines for the Diagnosis and Treatment of Acute Lung Injury/Acute Respiratory Distress Syndrome* and *Guidelines for Mechanical Ventilation in Patients with Acute Respiratory Distress Syndrome (Trial)*, including treatment of the primary disease (e.g., anti-infection), respiratory support (e.g., oxygen therapy, mechanical ventilation), and fluid replacement support. On this basis, participants in the low, medium, and high dose groups received a single intravenous infusion of BC-U001 at doses of 0.5×10^6^ cells/kg, 1.0×10^6^ cells/kg, and 1.5×10^6^ cells/kg, respectively, with an infusion rate of 1 ~ 3 mL/min, which could be adjusted appropriately according to the participant’s condition.

### Outcomes

#### Safety

Safety evaluation indicators included adverse events (AE), SAE, vital signs (body temperature, heart rate, respiratory rate, blood pressure), blood routine, blood biochemistry (liver function, kidney function, electrolytes, myocardial enzymes), coagulation function III, urine routine, 12-lead electrocardiogram, and cardiac ultrasound. AE were graded according to Common Terminology Criteria for Adverse Events (CTCAE) version 5.0 (grade 1 ~ 5), and the relationship between AE and the study drug was judged as definitely related, probably related, possibly unrelated, unrelated, or unevaluable. SAE referred to AE that resulted in death, were life-threatening, required hospitalization or prolonged hospitalization, caused disability, or led to congenital malformations, and were reported in a timely manner in accordance with regulatory requirements. SUSAR were serious adverse reactions whose nature and severity exceeded existing data (e.g., investigator’s brochure, product label) and were reported to relevant authorities, investigators, and IRBs as required.

#### Efficacy

Efficacy evaluation indicators included changes in PaO_2_/FiO_2_, number of deaths from all causes and all-cause mortality, changes in arterial blood gas analysis (pH, PaO_2_, PaCO_2_, lactic acid), LIS, SOFA, and APACHE II.

### Peripheral blood sample preparation

Peripheral blood samples from enrolled subjects must be collected during the screening/baseline period (Day -7 to Day 0), on Day 1 of the treatment period (pre-infusion and 24 hours post-infusion), follow-up period (Day 3, Day 7 ± 1, Day 14 ± 1, Day 28 ± 3), and upon early withdrawal (collected whenever possible). Blood samples should be collected from the antecubital vein or other suitable vein using aseptic technique. The volume of blood collected should be determined based on the specific test requirements. For complete blood count (CBC), coagulation factor III, lymphocyte subset analysis require collection in corresponding anticoagulant tubes (EDTA or sodium citrate), followed by gentle inversion 5 ~ 8 times to mix. Serum-related tests (blood biochemistry, inflammatory factors, etc.) require collection in non-anticoagulant tubes, followed by room temperature standing for 30 minutes to allow blood coagulation. Subsequently, centrifuge the non-anticoagulated tubes at 3000 rpm for 10 minutes to separate serum, then transfer serum to sterile EP tubes. All samples must be delivered to the designated laboratory within 2–4 hours post-collection. Serum samples unable to be delivered promptly must be stored at -20 °C. Anticoagulated whole blood samples must be refrigerated at 2 ~ 8 °C and protected from repeated freeze-thaw cycles. Each tube must be clearly labeled with the subject screening number, collection date, time point, test item, and sample type. Timely record collection time, blood volume, sample status, and submission time to ensure consistency with eCRF data.

### Detection of exploratory indicators

The testing methods for exploratory indicators in enrolled subjects are as follows: Seven immunoglobulins (Immunoglobulin G, IgG; Immunoglobulin A, IgA; Immunoglobulin M, IgM; Immunoglobulin E, IgE; Complement 3, C3; Complement 4, C4; Total Complement Hemolytic Activity, CH50) were measured using the immunoturbidimetric assay. Specific antibodies bind to corresponding immunoglobulins or complement components to form turbidity, and concentrations are calculated using a standard curve. Serum inflammatory-related factors (Interleukin-6, IL-6; Interleukin-8, IL-8; Interleukin-10, IL-10; Tumor Necrosis Factor-α, TNF-α; Epidermal Growth Factor, EGF) were measured using enzyme-linked immunosorbent assay (ELISA). Concentrations were determined by comparing enzyme-catalyzed substrate color development to a standard curve, based on antigen-antibody specific binding reactions. Total T lymphocytes (CD3^+^/CD19^-^), total B lymphocytes (CD3^-^/CD19^+^), total natural killer (NK) cells (CD3^-^/CD16^+^/CD56^+^) were analyzed using flow cytometry. Samples were stained with fluorescently labeled specific monoclonal antibodies, then analyzed via flow cytometer to assess cell surface marker expression, count, and calculate the number and percentage of each cell type. Erythrocyte Sedimentation Rate (ESR) was measured using the Weitman method, recording the distance red blood cells settled in anticoagulated blood over a specified time; C-Reactive Protein (CRP) was determined via immunoturbidimetry, converting the turbidity generated by antigen-antibody complex formation into concentration; Procalcitonin (PCT) is measured using electrochemiluminescence immunoassay or ELISA, detecting serum PCT levels based on the principle of antigen-antibody specific binding.

### Statistical analysis

Descriptive statistics were performed on baseline data for subjects in the low, medium, and high dose groups. Categorical variables were expressed as frequencies (percentages), with intergroup comparisons conducted using chi-square tests or Fisher’s exact probability test. Continuous variables were presented as mean ± standard deviation or median (interquartile range), with intergroup comparisons performed using one-way analysis of variance (for normally distributed data with homogeneity of variance) or Kruskal-Wallis H test (for non-normal distribution or unequal variances). The incidence of AE during treatment was described as frequencies (percentages) for each group of subjects, including overall incidence and AE incidence by system organ classification. Intergroup incidence comparisons were performed using the chi-square test or Fisher’s exact test. Intergroup distribution comparisons of AE severity (graded according to CTCAE version 5.0) were conducted using the rank-sum test for ordered categorical data. The Kaplan-Meier method was used to calculate the survival rates without SAEs for the three patient groups. The Log-rank test was employed to compare differences between groups (with Bonferroni correction to control for Type I errors). For PaO_2_/FiO_2_, arterial blood gas parameters, LIS, SOFA, and APACHE II scores, comparisons of differences within each group before and after treatment (baseline vs. post-treatment time points) were performed using paired t-tests (for normally distributed data) or Wilcoxon signed-rank tests (for non-normally distributed data). The change value between pre- and post-treatment (post-treatment indicator value - baseline value) serves as the effect indicator. Intergroup comparisons employ one-way ANOVA (for normally distributed data with homogeneity of variance) or the Kruskal-Wallis H test (for non-normally distributed data or heterogeneous variance). If statistically significant differences exist, pairwise comparisons are further conducted (using Bonferroni correction or Dunn’s test). Simultaneously, the change rate relative to baseline and 95% confidence intervals for each indicator at different time points post-treatment were calculated for each group, enabling trend analysis between groups. Calculate the all-cause mortality rate for each group of subjects during the follow-up period. Intergroup comparisons were performed using Fisher’s exact probability test. Survival curves (Kaplan-Meier method) were plotted, and intergroup survival differences were assessed using the log-rank test. For immunoglobulin levels and serum inflammatory cytokines, paired t-tests or Wilcoxon signed-rank tests were used to assess differences within groups before and after treatment. Between-group comparisons of change values employed one-way ANOVA or Kruskal-Wallis H tests. Data underwent log transformation (where skewness was severe) prior to statistical analysis to enhance test power. For lymphocyte subsets, ESR, CRP, and PCT, the statistical methods are the same as those used for immunoglobulin and inflammatory factor analysis. Among these, percentage data for lymphocyte subsets must undergo arcsin transformation before parametric testing (if the conditions for normal distribution are met). All statistical analyses were performed using SAS^®^ 9.4 software. A two-tailed test was applied with a significance level of α = 0.05. The *P < 0.05* was considered statistically significant.

## Data Availability

The raw data supporting the conclusions of this article will be made available by the authors, without undue reservation.
