# Supplementary material for: The safety and efficacy of human umbilical cord mesenchymal stem cell for acute respiratory distress syndrome: an open-label and multicenter phase 1 clinical trial
Source: Front Immunol. 2026 Jun 10;17:1848989. doi: 10.3389/fimmu.2026.1848989 (PMC13290605; doi:10.3389/fimmu.2026.1848989)
Supplement: Supplementary file 1 [file DataSheet1.docx]

**Supplementary information for**

**The Safety And Efficacy of** **Human Umbilical Cord Mesenchymal Stem Cell for Acute Respiratory Distress Syndrome: An Open-label And Multicenter Phase 1 Clinical Trial**

Qinggang Ge ^1#^, Libo Zheng ^2#^, Xizeng Cui ^2#^, Man Zhao ^1^, Chao Li ^1^, Zhiling Zhao ^1^, Zongyu Wang ^1^, Qiang Zhang ^1^, Mai Shi ^1^, Yuxuan Li ^1^, Jianan Zhang ^1^, Yixian Qiao ^3^, Senhao Wei ^4^, Ning Shen ^3^, Haomiao Long ^2^, Yongjun Liu ^2*^, Jie Qiao ^5*^

1.Department of Intensive Care Medicine, Peking University Third Hospital, Beijing 100191,China

2.Stem Cell Biology and Regenerative Medicine Institution, Yi-Chuang Institute of Bio-Industry, Beijing 100176, Beijing, China.

3.Department of Intensive Care Medicine, Peking University Third Hospital, Beijing 100191,China

4.Department of Pulmonary and Critical Care Medicine, Peking University Third Hospital, Beijing 100191,China

5.State Key Laboratory of Female Fertility Promotion, Center for Reproductive Medicine, Department of Obstetrics and Gynecology, Peking University Third Hospital, Beijing 100191, Beijing, China.

**# These authors made equal contributions.**

***** **Correspondence to:**

Yongjun Liu, Stem Cell Biology and Regenerative Medicine Institution, Yi-Chuang Institute of Bio-Industry, Beijing 100176, Beijing, China.

E-mail: [liuyongjun@blswinc.com](mailto:liuyongjun@blswinc.com)

Jie Qiao, State Key Laboratory of Female Fertility Promotion, Center for Reproductive Medicine, Department of Obstetrics and Gynecology, Peking University Third Hospital, Beijing 100191, Beijing China.

E-mail: [jie.qiao@263.net](mailto:jie.qiao@263.net)

**Study procedures**

The study was divided into three phases: screening/baseline phase (days -7 ~ 0), treatment phase (days 1 ~ 3), and follow-up phase (days 4 ~ 28). During the screening phase, participants underwent a comprehensive assessment including medical history collection, physical examination, vital signs measurement, and laboratory tests (blood routine, blood biochemistry, coagulation function III, arterial blood gas analysis, immunoglobulin seven items, serum inflammatory factor detection, lymphocyte subsets, ESR, CRP, PCT, urine routine, 12-lead electrocardiogram, cardiac ultrasound, chest X-ray, urine pregnancy test [for females], hepatitis B five items, hepatitis C antibody, syphilis, AIDS, γ-interferon release test, and tumor marker detection. LIS, SOFA, and APACHE II were also evaluated to confirm eligibility.

During the treatment phase (day 1), after completing the baseline assessment, participants received the corresponding dose of BC-U001 infusion, with vital signs monitored at 1 hour and 6 hours after infusion, and adverse events (AE) and concomitant medications recorded. At 24 hours after infusion, arterial blood gas analysis, serum inflammatory factor detection, lymphocyte subset analysis, and 12-lead electrocardiogram were re-examined. On day 2, vital signs, AE, and concomitant medications were recorded; on day 3, a comprehensive re-examination (same as the screening phase, excluding infectious disease screening and tumor marker detection) and score evaluation (LIS, SOFA, APACHE II) were performed, and DLT was assessed.

During the follow-up phase, participants were followed up at days 7 ± 1, 14 ± 1, and 28 ± 3, with physical examination, vital sign monitoring, AE and concomitant medication recording, laboratory re-examination (same as day 3), and score evaluation completed at each follow-up time point to monitor long-term safety and efficacy.

**Duration of the trial**

Follow-up for each study participant.


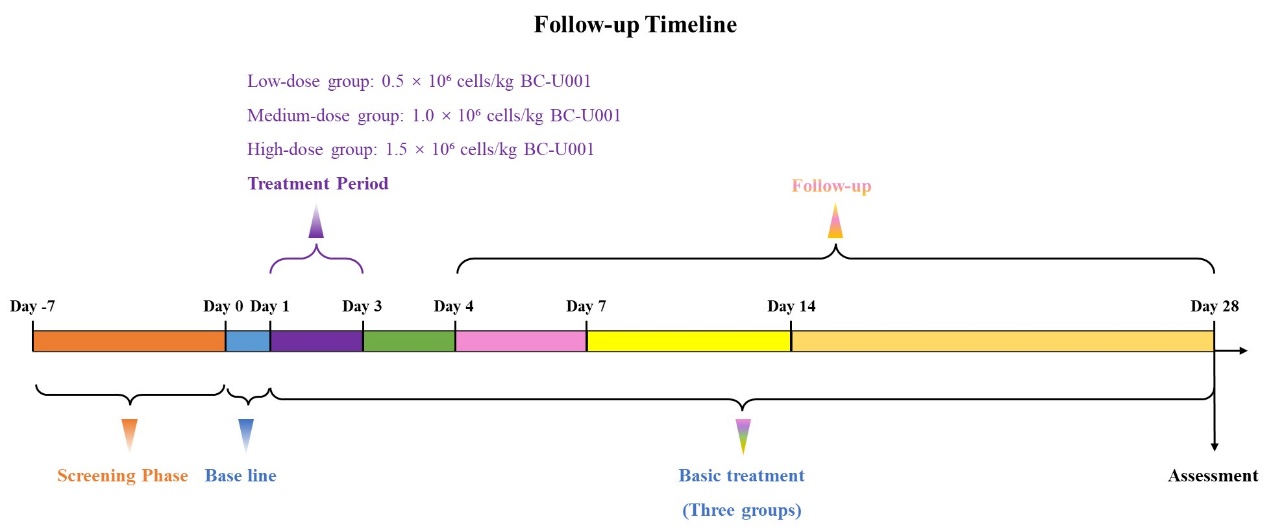


**Supplementary Figure 1.** **Follow-up timeline.** Each subject's investigation lasted 28 days after the screening period. At least 10 follow-up visits were conducted for individual: including the screening, baseline, treatment, and follow-up.

**Supporting Results**


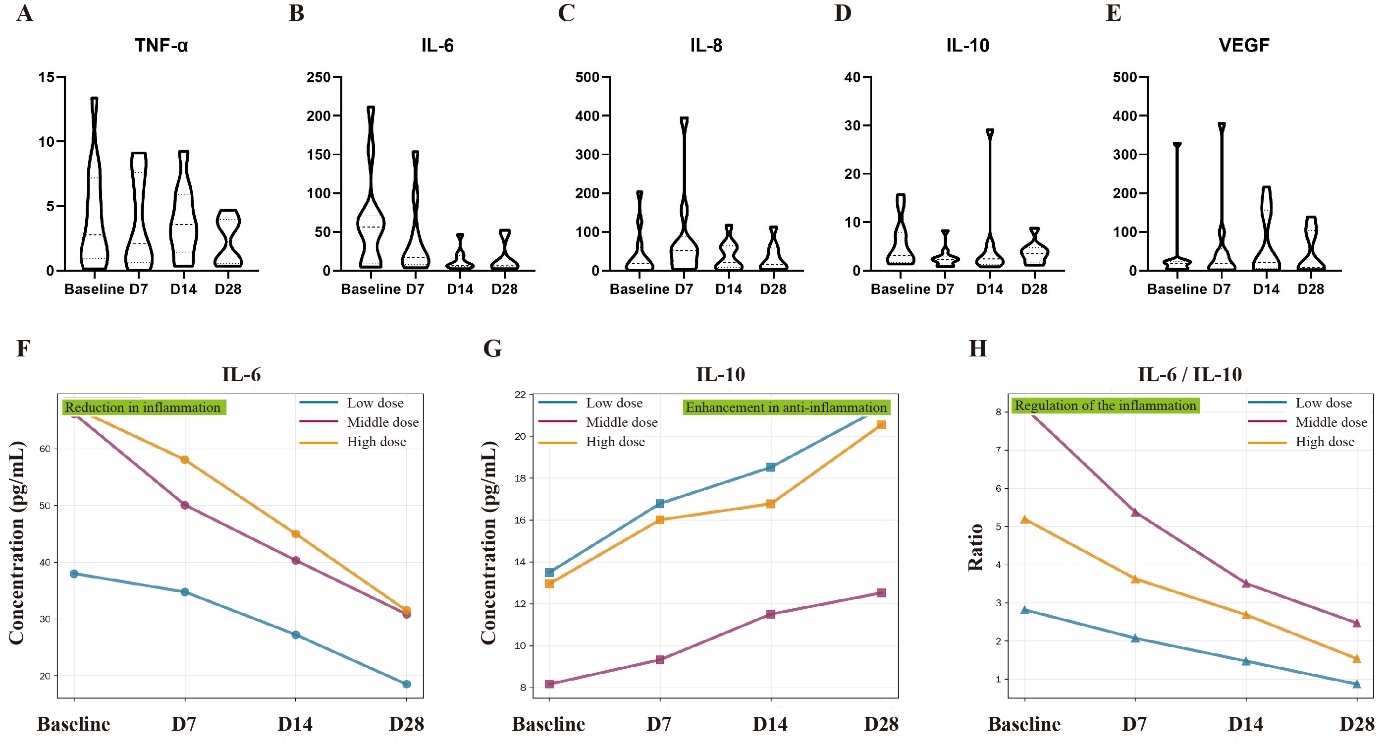


**Supplementary Figure 2. Changes in serum cytokines.** A-E: The Violin plot of concentration of TNF-α, IL-6, IL-8, IL-10, and EGF in all subjects except for dropouts from baseline to Day 14, separately. F: Changes in serum IL-6 levels among the three groups at each visit. G: Changes in serum IL-10 levels among the three groups at each visit. H: Changes in ratio of IL-6/IL-10 among the three groups at each visit.


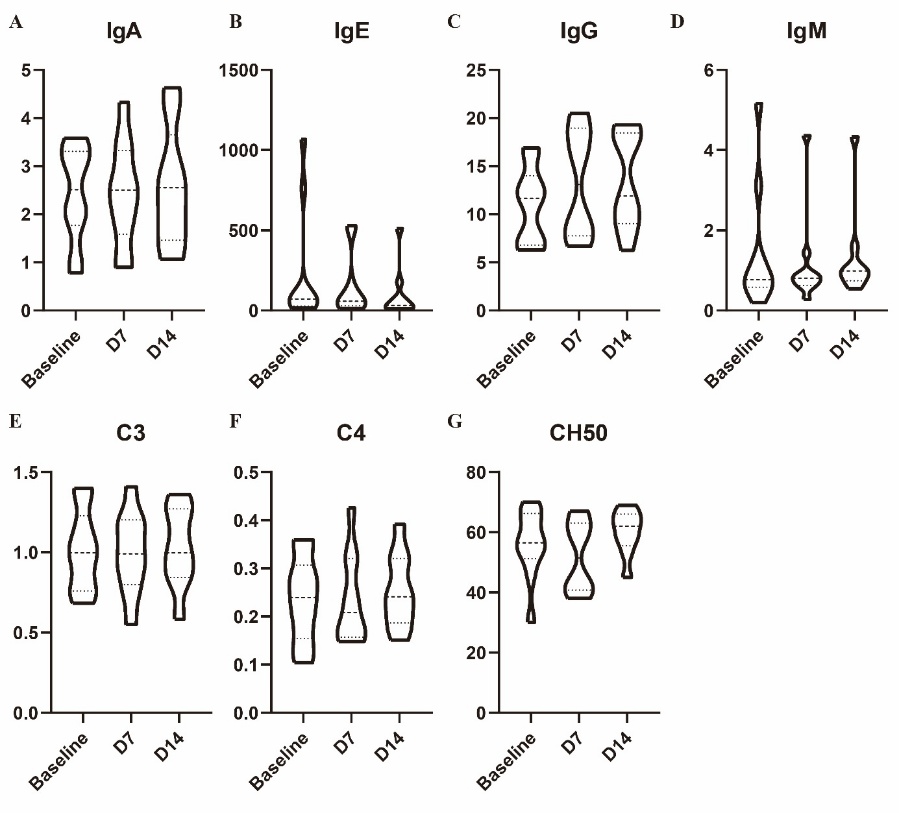


**Supplementary Figure 3. Changes in immunoglobulins and complements.** A-G: The Violin plot of concentration of IgA, IgE, IgG, IgM, C3, C4 and CH50 in all subjects except for dropouts from baseline to Day 14, separately.

**Supplementary Table 1.** **Log-rank test for survival differences among three groups**

|  | **Log-rank χ²** | **P** |
| --- | --- | --- |
| Low vs. Middle | 0.006 | 0.937 |
| Low vs. High | 0.750 | 0.386 |
| Middle vs. High | 0.600 | 0.439 |

**Supplementary Table 2.** **Improvements in oxygenation index among the three groups of subjects**

| **Indicator** | **Low dose** | **Middle dose** | **High dose** | ***Intergroup P-value*** |
| --- | --- | --- | --- | --- |
| **PaO₂/FiO₂, mmHg** |  |  |  | *0.236* |
| **Base line** | 222.66 ± 53.24 | 173.48 ± 11.96 | 171.37 ± 44.33 |  |
| **Day 28** | 322.73 ± 76.39 | 250.91 ± 15.39 | 249.34 ± 55.96 |  |
| **Δ** | +100.07±24.7 | +77.44 ± 5.57 | +77.97 ± 12.30 |  |
| ***Intragroup P-value*** | *0.125* | ***< 0.001*** | *0.250* |  |

**Supplementary Table 3.** **Improvements of** **PaO₂ among the three groups of subjects**

| **Indicator** | **Low dose** | **Middle dose** | **High dose** | **Intergroup P-value** |
| --- | --- | --- | --- | --- |
| **PaO₂ mmHg** |  |  |  | *0.486* |
| **Base line** | 71.06 ± 13.92 | 64.22 ± 8.94 | 80.46 ± 4.59 |  |
| **Day 28** | 93.23 ± 23.14 | 86.10 ± 14.06 | 107.83±8.22 |  |
| **Δ** | +22.16 | +21.88 | +27.37 |  |
| ***Intragroup P-value*** | *0.125* | ***0.002*** | *0.250* |  |

**Supplementary Table 4.** **Changes of lactic acid among the three groups of subjects**

| **Indicator** | **Low dose** | **Middle dose** | **High dose** | ***Intergroup P-value*** |
| --- | --- | --- | --- | --- |
| **Lac, mmol/L** |  |  |  | *0.177* |
| **Base line** | 1.60 ± 0.50 | 1.97 ± 0.61 | 2.42 ± 0.83 |  |
| **Day 28** | 0.98 ± 0.29 | 1.18 ± 0.37 | 1.48 ± 0.48 |  |
| **Δ** | -0.62 | -0.79 | -0.94 |  |
| ***Intragroup P-value*** | *0.125* | ***0.002*** | *0.250* |  |

**Supplementary Table 5.** **Changes of LIS among the three groups of subjects**

| **Indicator** | **Low dose** | **Middle dose** | **High dose** | ***Intergroup P-value*** |
| --- | --- | --- | --- | --- |
|  |  |  |  | *0.055* |
| **Base line** | 1.93 ± 0.31 | 1.87 ± 0.44 | 2.53 ± 0.32 |  |
| **Day 28** | 1.05 ± 0.20 | 1.04 ± 0.26 | 1.37 ± 0.16 |  |
| **Δ** | -0.88 | -0.82 | -1.16 |  |
| ***Intragroup P-value*** | *0.125* | ***< 0.001*** | *0.250* |  |

**Supplementary Table 6.** **Changes of SOFA score among the three groups of subjects**

| **Indicator** | **Low dose** | **Middle dose** | **High dose** | ***Intergroup P-value*** |
| --- | --- | --- | --- | --- |
|  |  |  |  | *0.157* |
| **Base line** | 6.11±1.15 | 5.46 ± 1.86 | 7.59 ± 0.28 |  |
| **Day 28** | 3.55 ± 0.75 | 3.38 ± 1.22 | 4.57 ± 0.16 |  |
| **Δ** | -2.57 | -2.087 | -3.02 |  |
| ***Intragroup P-value*** | *0.125* | ***0.002*** | *0.250* |  |

**Supplementary Table 7.** **Changes of** **APACHE Ⅱ score among the three groups of subjects**

| **Indicator** | **Low dose** | **Middle dose** | **High dose** | ***Intergroup P-value*** |
| --- | --- | --- | --- | --- |
|  |  |  |  | *0.090* |
| **Base line** | 14.34 ± 1.79 | 19.63 ± 4.09 | 13.69 ± 3.70 |  |
| **Day 28** | 9.53 ± 1.65 | 12.55 ± 2.41 | 9.16±3.09 |  |
| **Δ** | -4.80 | -7.08 | -4.53 |  |
| ***Intragroup P-value*** | *0.125* | ***< 0.001*** | *0.250* |  |

**Supplementary Table 8.** **Changes in serum IL-6 and IL-10 (D28 V.S. Baseline)**

| **Indicator** | **Low dose** | **Middle dose** | **High dose** |
| --- | --- | --- | --- |
| **IL-6** | 38.01±28.93→18.52±14.21  (-51.3%) | 66.18±24.28→30.87±11.56  (-53.4%) | 67.26±32.49→31.53±15.40  (-53.1%) |
| **IL-10** | 13.49±3.38→21.37±6.26  (+58.4%) | 8.15±3.46→12.52±5.64  (+53.5%) | 12.95±2.89→20.55±4.75  (+58.7%) |
| **IL-6 / IL-10** | 2.81→0.87  (-69.0%) | 8.12→2.46  (-69.7%) | 5.20→1.53  (-70.6%) |

**Characterization and quality control of BC-U001**

The flow cytometry analysis (Panel A) confirmed the immunophenotype of BC-U001 cells in accordance with the International Society for Cellular Therapy (ISCT) criteria. The cells exhibited high expression of positive MSC markers CD73 (99.9 %), CD90 (100.0 %), and CD105 (99.93 %), while showing negligible expression of negative markers CD19 (0.72 %), CD34 (0.17 %), CD31 (0.35 %), CD11b (0.51 %), CD45 (0.22 %), and HLA-DR (0.28 %) (Supplementary Figure 4. A). Isotype controls (Mouse IgG1 FITC/PE) confirmed the specificity of the staining. Trypan blue staining (Panel B) demonstrated high cell quality with a viability of 98.58 %, a total cell concentration of 5.49 × 10^7^/mL, and an aggregation rate of only 4.27 % (Supplementary Figure 4. B). The standard calibration curve for the soluble tumor necrosis factor receptor 1 (sTNFR1) potency assay (Panel C) showed excellent linearity (R^2^ = 0.9982) (Supplementary Figure 4. C), validating the biological potency measurement system for BC-U001 cells.


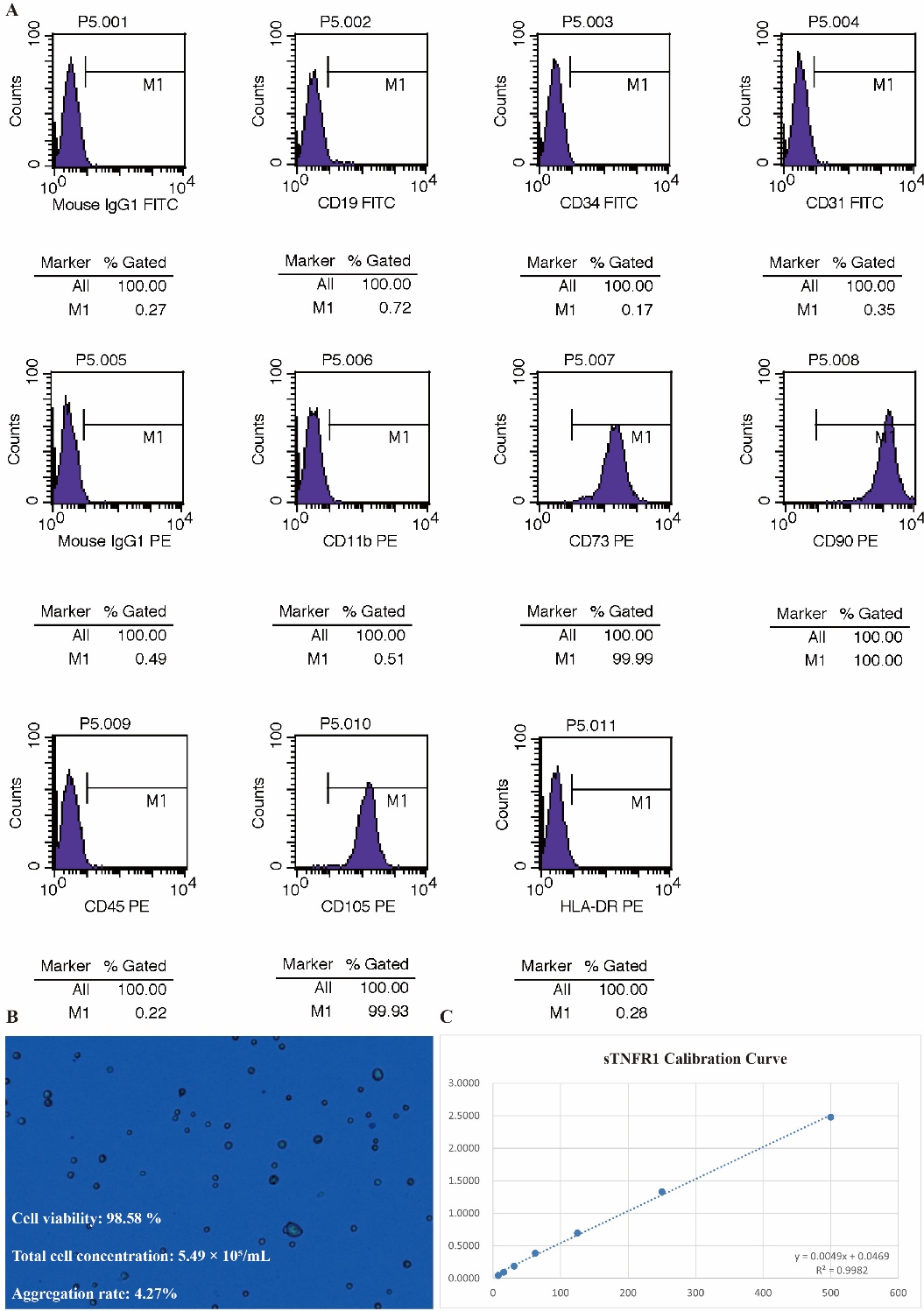
**Supplementary Figure 4. Changes in immunoglobulins and complements.** A: Immunophenotypic analysis of BC-U001 cells by flow cytometry. Cells were stained with antibodies against MSC positive markers (CD73, CD90, CD105) and negative markers (CD19, CD34, CD31, CD11b, CD45, HLA-DR). Isotype-matched antibodies (Mouse IgG1 FITC/PE) were used as controls. The percentage of cells positive for each marker (within gate M1) is indicated. B: Cell viability, concentration, and aggregation rate of BC-U001 cells determined by trypan blue staining. The measured values are: cell viability 98.58 %, total cell concentration 5.49 × 10^7^/mL, and aggregation rate 4.27 %. C: Calibration curve for the soluble tumor necrosis factor receptor 1 (sTNFR1) potency assay. The linear regression equation (y = 0.0049x + 0.0469, R^2^ = 0.9982) demonstrates high linearity, validating the method for assessing the biological potency of BC-U001 cells.
